# The altered TBI fecal microbiome is stable and functionally distinct

**DOI:** 10.3389/fnmol.2024.1341808

**Published:** 2024-03-13

**Authors:** Richard B. Pyles, Aaron L. Miller, Randall J. Urban, Melinda Sheffield-Moore, Traver J. Wright, Carrie A. Maxwell, Kathleen M. Randolph, Christopher P. Danesi, Kristen A. McGovern, Jayson Vargas, Peyton Armstrong, Lisa Kreber, Giuliana Cumpa, Kevin Randall, Melissa Morrison, Brent E. Masel

**Affiliations:** ^1^Department of Pediatrics, The University of Texas Medical Branch, Galveston, TX, United States; ^2^Department of Internal Medicine, The University of Texas Medical Branch, Galveston, TX, United States; ^3^Department of Microbiology and Immunology, The University of Texas Medical Branch, Galveston, TX, United States; ^4^John Sealy School of Medicine, The University of Texas Medical Branch, Galveston, TX, United States; ^5^Centre for Neuro Skills, Bakersfield, CA, United States; ^6^Department of Neurology, The University of Texas Medical Branch, Galveston, TX, United States

**Keywords:** traumatic brain injury, microbiome, fecal microbiome, metatranscriptome, BIAFAC

## Abstract

**Introduction:**

Patients who suffer a traumatic brain injury (TBI) often experience chronic and sometimes debilitating sequelae. Recent reports have illustrated both acute and long-term dysbiosis of the gastrointestinal microbiome with significant alterations in composition and predicted functional consequences.

**Methods:**

Working with participants from past research, metagenomic stability of the TBI- associated fecal microbiome (FMB) was evaluated by custom qPCR array comparing a fecal sample from 2015 to one collected in 2020. Metatranscriptomics identified differently expressed bacterial genes and biochemical pathways in the TBI FMB. Microbiota that contributed the largest RNA amounts identified a set of core bacteria most responsible for functional consequences of the TBI FMB.

**Results:**

A remarkably stable FMB metagenome with significant similarity (two-tail Spearman nonparametric correlation *p* < 0.001) was observed between 2015 and 2020 fecal samples from subjects with TBI. Comparing the 2020 TBI FMB metagenome to FMBs from healthy controls confirmed and extended the dysbiotic genera and species. Abundance differences between average TBI and healthy FMBs revealed *Bacteroides caccae, B*. *uniformis, Blautia* spp., *Collinsella* spp., *Dialister* spp., and Ordoribacter spp. were significantly different. Functionally, the *Parabacteroides* genus contributed the highest percentage of RNA sequences in control FMBs followed by the *Bacteroides* genus as the second highest contributor. In the TBI FMB, the *Corynebacterium* genus contributed the most RNA followed by the *Alistipes* genus. *Corynebacterium* and *Pseudomonas* were distinct in the top 10 contributing genera in the TBI FMB while *Parabacteroides* and *Ruminococcus* were unique to the top 10 in controls. Comparing RNA profiles, TBI samples had ∼1.5 fold more expressed genes with almost 700 differently expressed genes (DEGs) mapped to over 100 bacterial species. Bioinformatic analysis associated DEGs with pathways led identifying 311 functions in the average TBI FMB profile and 264 in the controls. By average profile comparison, 30 pathways had significantly different abundance (*p* < 0.05, *t*-test) or were detected in >80% of the samples in only one of the cohorts (binary distinction).

**Discussion:**

Functional differences between TBI and healthy control FMBs included amino acid metabolism, energy and carbon source usage, fatty acid metabolism, bacterial cell wall component production and nucleic acid synthesis and processing pathways. Together these data shed light on the functional consequences of the dysbiotic TBI FMB decades after injury.

## Introduction

Traumatic brain injury (TBI) is remarkably common with an estimated 600 TBI-related hospitalizations and more than 170 TBI-related deaths each day in the United States (Peterson and Thomas, 2021; Read, n.d.). In an Ohio based study, 1 in 5 adults reported a TBI with loss of consciousness and for 10% of respondents the TBI occurred before they were 15 years old (Corrigan et al., 2018). TBI are associated with long-term health issues including increased risk of stroke (Esterov et al., 2023), early onset dementia (Bellomo et al., 2022), depression (Lambert et al., 2022), and decreased olfaction (Tai et al., 2022). Post-TBI sequelae frequently include fatigue and altered cognition that compromise quality of life and productivity as described by the brain injury associated fatigue and altered cognition syndrome (BIAFAC) (Yuen et al., 2020). BIAFAC is characterized clinically by abnormal growth hormone (GH) levels after glucagon stimulation and hypoaminoacidemia (Børsheim et al., 2007; Durham et al., 2017; Mossberg et al., 2017; Wright et al., 2020; Yuen et al., 2020) raising the possibility that BIAFAC and other TBI-related sequelae may be caused by a dysbiotic gastrointestinal microbiome. We have studied the potential role of the microbial community structure in chronic TBI through fecal sampling followed by bacterial 16S rDNA, *tuf* gene and shot-gun, whole genome sequencing (WGS). These data directed creation of a customized qPCR array to provide absolute abundance of selected microbiota (Urban et al., 2020).

In clinical samples and animal models the fecal microbiome (FMB) is significantly altered soon after even mild TBI, and develops into chronic dysbiosis in some patients (Treangen et al., 2018; Nicholson et al., 2019; Brenner et al., 2020; Pathare et al., 2020; Urban et al., 2020; Yuen et al., 2020; Opeyemi et al., 2021; Aghakhani, 2022; Soriano et al., 2022). Previously reported WGS data from fecal samples from patients who had a moderate to severe TBI predicted functional alterations in amino acid (AA) metabolism, short chain fatty acid (SCFA) biosynthesis/oxidation and communication with the nervous system including the pituitary (Urban et al., 2020). Dysbiotic FMB communities are increasingly associated with a variety of other neurocognitive and neuropathological conditions (Sochocka et al., 2019; Elfil et al., 2020; Johnson et al., 2020; Sun et al., 2020; Alharthi et al., 2022; Dinan and Dinan, 2022). Comparing the dysbiotic FMBs from individuals suffering with Parkinson’s disease, autism spectrum disorder, Alzheimer’s disease, and other pathologies has revealed an interesting list of bacterial genera that may produce detrimental metabolites and/or metabolize critical dietary components including essential AAs. This may lead to deficiencies that have not yet understood consequences on nervous system function.

It appears that the TBI FMB may cause similar impacts on the gut-brain axis (Sundman et al., 2017). We previously reported that the FMB communities in samples from participants with moderate to severe, debilitating TBI that occurred, on average, >20 years prior, were distinct from subjects without TBI in two care facilities located in California and Texas (Urban et al., 2020). Our findings illustrated changes in abundance of species in the *Bacteroides* and *Prevotella* genera; consistent with findings reported more recently for other neuropathologies (Rogers et al., 2016; Brenner et al., 2020; George et al., 2021; Hanscom et al., 2021). Our WGS analysis predicted substantial changes in molecular functions of the FMB communities in TBI subjects relative to control subjects including AA metabolism, SCFA metabolism, and energy utilization (Urban et al., 2020).

Our clinical research and the field broadly support continued investigation of the bi-directional signaling between the gut and brain and determination of whether FMB dysbiosis is sustained following TBI. FMB stability in large human cohorts has been reported across time spans of >4 years (Faith et al., 2013; Chen et al., 2021; Frost et al., 2021). Collective data indicated that FMBs with greater alpha-diversity had higher stability and that several bacterial genera including *Bacteroides* were among the most stable over time. To address our theory that TBI FMB dysbiosis is similarly stable, we analyzed the TBI FMB community profile 5 years after our initial study in a small cohort of past participants with moderate to severe debilitating TBI living continuously in the Centre for Neuro Skills (CNS) facility (Bakersfield, CA). In this first kinetic study of TBI FMBs, analysis by qPCR array confirmed the stability of the TBI FMB metagenomic community structure over the 5-year sampling window.

To better understand the molecular consequences of the shifted metagenome, we also completed bioinformatic analyses of the metatranscriptome in the TBI FMB samples and compared that profile to healthy controls. This novel approach identified microbiota that were most contributory to the TBI FMB metatranscriptome and those molecular pathways that were significantly different between the groups. The results illustrated that some metagenomically identified bacteria did not contribute measurable RNA while other novel bacteria were recognized as important contributors but were not noted as having significantly altered abundance in past qPCR metagenomic comparisons. Application of these newer technologies and bioinformatic workflows will focus future study designs on only those bacteria that contribute to functional distinctions. Differences in expression of microbial biochemical pathways confirmed and extended prior WGS predictions including differences in specific AA metabolism pathways, glycolysis and altered energy usage, and revealed impacts on nucleotide metabolism and metabolism of lipids and SCFAs. Collectively the data add confidence that subjects with moderate to severe TBI have stable dysbiotic FMB community structures with distinct functional profiles that may contribute to chronic sequelae and increased risk for pathology. A greater understanding of these alterations is critical to design effective interventions to improve quality of life for individuals suffering long-term consequences of TBI.

## Materials and methods

### Ethics statement

All subjects provided written informed consent before self or assisted collection of a single fecal sample. All procedures were approved by the Advarra IRB (Pro00027812) for subjects at the CNS facility (Bakersfield, CA) or by the University of Texas Medical Branch (UTMB) IRB for control samples collected in Galveston County, TX. Samples were assigned a unique study identification number to minimize personal information exposure. Limited metadata were connected to each study ID (Table 1).

**TABLE 1:** Study cohort demographics.

Group	Age	Height (cm)	Weight (kg)	BMI	Ambulatory	Time since injury (months)
TBI 1	41	180.34	88.08	27.1	Y	312
TBI 2	62	170.18	82.91	28.6	Y	264
TBI 3	53	180.34	65.77	20.2	N	324
TBI 4	44	165.1	74.93	27.5	Y	301
TBI 5	56	193.04	92.8	24.9	Y	266
**TBI AVG**	**51.2 (8.6)**	**177.8 (10.8)**	**80.9 (10.7)**	**25.7 (3.3)**	**4Y/1N**	**293.4 (27.2)**
Control 1	59	183.8	121.4	35.9	Y	N/A
Control 2	57	185.5	112.2	32.6	Y	N/A
Control 3	51	179	90	28.09	Y	N/A
Control 4	60	183.4	98.2	29.2	Y	N/A
Control 5	44	190.5	99.7	27.5	Y	N/A
**Control AVG**	**54.2 (6.7)**	**184.4 (4.2)**	**104.3 (12.4)**	**30.6 (3.5)**	**5Y**	**N/A**

Average values for each group are shown in bold text with (SDEV) indicated. The controls showed no significant average differences compared to the TBI subject cohort (Mann–Whitney *t*-tests).

### Subjects

Study participants with moderate to severe TBI were enrolled based on participation in our previous study (Urban et al., 2020). Five continual residents of the CNS facility who previously donated a fecal sample for analysis in 2015 (Urban et al., 2020) agreed to participate and were consented under a new IRB protocol. They resided continuously in the facility since the 2015 sampling. Five male age- and BMI-matched healthy control subjects without a history of TBI were recruited through advertisement and enrolled from the Galveston, TX area. After obtaining informed consent, participants were provided fecal sampling kits and were instructed on the collection technique. Single fecal samples were collected at the end of 2020 prior to any COVID-19 diagnosis in the CNS facility.

### Fecal microbiota DNA and RNA preparation

As previously described (Urban et al., 2020), fecal samples were collected into OMNIgene GUT kit OMR-200 (DNA Genotek, Ottawa, ON, Canada) and delivered within 48 h to UTMB (Galveston-control) or stored at −80°C until shipped on dry ice (CNS-TBI). For processing, samples were thawed and mixed with 3 ml of sterile PBS, vigorously vortexed, and 50 μl of the resulting slurry was mixed with 200 μl PM1 solution (Qiagen) within a 0.1 glass Powerbead tube (Qiagen). The remaining material was archived at −80°C. Each sample was heated to 55°C for 30 min and then bead beaten (5 min at 30 Hz; Qiagen TissueLyser LT). The liquid fraction (clarified at 13K × g for 1 min) was mixed with inhibitor reduction solution (IRS; Qiagen), incubated at 4°C for 5 min. Nucleic acid (NA) was extracted using a MagNAPure96 system and the DNA and viral NA small volume kit (Roche Applied Science, Indianapolis, IN, USA) or the MagNAPure96 cellular RNA large volume kit (Roche). DNAs were analyzed for quality using qPCR (Urban et al., 2020). Extracted RNA was quality checked (16s rRNA and human GAPDH abundance) before NGS library synthesis. Residual NA was stored at −80°C. Negative quality control samples were processed in parallel.

### Fecal metagenomic stability comparisons

DNA from each of the five fecal samples collected in 2020 were subjected to our custom TBI FMB qPCR array (Urban et al., 2020) that included quantification of total bacterial DNA and human DNA. FMB metagenomic profiles were normalized to the amount of total bacterial DNA recovered to allow comparison to the profiles created in 2015 to minimize technical impacts created by slight changes in the extraction kit and fecal sampling kits employed. All samples from 2015 and 2020 surpassed the quality cutoffs (>1E7 bacterial genomes/μl of recovered DNA). Novel bacterial target qPCR assays are described in Supplementary Table 1. Normalized metagenomic profiles were subjected to similarity and correlation analyses as described below.

### Fecal metatranscriptomics and bioinformatics

Bacterial and human ribosomal RNAs (rRNA) were removed from the recovered RNA using the RiboMinus Bacteria 2.0 Transcriptome Isolation and RiboMinus Eukaryotic Kits v2 (ThermoFisher, Waltham, MA, USA). Target probes were mixed in a 4:1 ratio of bacteria to human and used following the manufacturer’s protocol. Libraries were prepared with a NEBNext Ultra II RNA Library kit (New England Biolabs, Ipswich, MA, USA). Libraries were quantified, pooled and sequenced with the paired-end 75 base protocol on a NextSeq 550 using the High-Output flow cell (Illumina Inc., San Diego, CA, USA). RNA-negative water samples were analyzed to confirm no environmental contaminants were present.

FASTQ files for bacterial metatranscriptomics with human host reads removed were analyzed with Galaxy Europe web-based bioinformatics^1^ using the established ASaiM workflow (Baker et al., 2016; Batut et al., 2018; Mehta et al., 2021). Briefly, the sequencing data were processed to remove low quality and adapter sequences (Martin, 2011). All samples surpassed the minimum of 5E6 high quality reads required for inclusion. A community profile was extracted with the assignment of relative abundance to taxonomic levels using MetaPhlAn2 (Truong et al., 2015). The sequences were sorted to categorize rRNA reads from functional reads needed for gene and pathway annotation (Kopylova et al., 2012) and then used to derive functional information using the HUMAnN2 pipeline (Abubucker et al., 2012). The results of both MetaPhlAN2 and HUMAnN2 workflows were subsequently combined to associate taxonomic and functional information corresponding to relative abundance of microbiota identified from the community profile. Raw data files are available (BioProject PRJNA1005267).

Pathway abundance levels for each specific genera/species were sum totaled using pivot tables (Excel). Totals were normalized via conversion to relative abundance (% or total) then compared using unpaired *t*-tests (*p* ≤ 0.05) without correction assuming a <0.05 *p*-value for significance. Binary comparison was carried out by comparing the presence or absence of pathways between groups (±80% Community, ±30% *Bacteroides*-specific, Control vs. TBI). Microbiota at the genus and species levels contributing to pathways with statistically significant differences in expression were compared via relative abundance between control and TBI cohorts. Clustering comparison and visualization of the data was accomplished using PCA and associated heat-maps (Metsalu and Vilo, 2015).

### Statistical analysis

For metagenomic comparisons, Pearson’s correlation was calculated in Excel. After normalization to total bacterial genomic load in each sample (16S rDNA copies), two-tail Spearman nonparametric correlation was performed and plotted (Prism v9.5.1; GraphPad, Boston, MA, USA). To compare normalized abundance of genomic copies between the TBI and control cohorts, paired and unpaired multiple *t*-test analyses were performed (Prism) as well as Fisher’s exact test calculations for binary comparisons.^2^ For qPCR absolute abundance data comparisons, statistical significance was determined using multiple *t*-tests via the Holm-Sidak method (Prism). Each qPCR target was analyzed individually, without assumption of a consistent SD. A *p*-value of <0.05 was considered significant. Clustering analysis was completed using Morpheus web-based software (Broad Institute, Cambridge, MA, USA) and ClustVis (Metsalu and Vilo, 2015). RNA data analyses are described in the previous section.

## Results

### Study cohort

This study was made possible by the willingness of five subjects living in the CNS facility (Bakersfield, CA) to participate in a follow up FMB analysis. Two control subjects had COVID-19 more than 6 months prior to sampling and did not report any lingering effects. None of the participants had any illness or antibiotic use in the 3 months prior to collection. Cohort demographics are summarized in Table 1. There were no significant differences in the general characteristics of the two groups although the average age and BMI were slightly higher in the controls.

### TBI FMB metagenome profiles are stable over time

**FIGURE 1:**
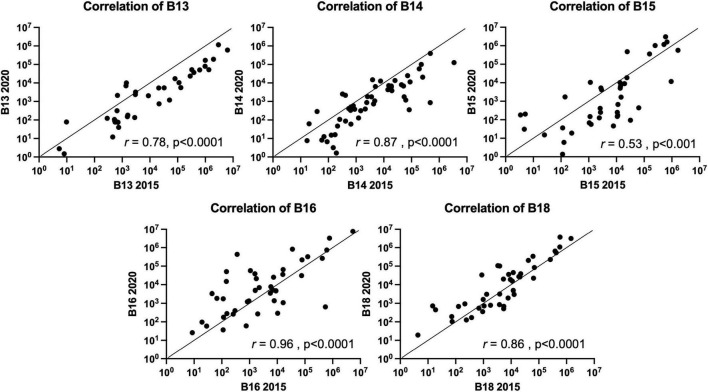
Spearman nonparametric correlation (two-tail) plots of the FMB metagenomic profiles of five subjects with TBI. Similarity indices (Spearman *rho* value) based on the results from the 96 target custom qPCR array for TBI FMB are shown (*r*). The *x*-axis depicts the original profile (2015) and the *y*-axis shows the 2020 sample distribution. For hypothesis testing the *p*-value was set to 0.05.

Stability of the FMB in individuals with a TBI received on average 24.5 years prior to sampling was evaluated by qPCR array (Urban et al., 2020). In this follow-up study, five participants from our original study (2015) provided a fecal sample roughly 5 years later. Each sample was evaluated for changes in the FMB community profile based on the targets included in our custom TBI FMB qPCR array (Urban et al., 2020) as well as additional bacterial targets identified in ongoing research. Pearson’s correlations on each absolute abundance pairing (2015 versus 2020) without normalization showed >99% similarity for each FMB pair. Because of the variation in total 16S rDNA abundance and changes in the collection materials over the 5 years, each profile was normalized to the total 16S rDNA content (the lowest 16S rDNA copy number was 7.5E7 for TBI and 2.58E7 for control FMB samples) and then correlation matrices for each pair were generated. The plotted correlations showed remarkable consistency with *rho* values (two-tail Spearman nonparametric correlation) ranging from 0.53 to 0.96 (Figure 1). By this comparison, all five pairs were significantly similar (*p* > 0.001) indicating minimal changes to previously reported FMB metagenomic community profiles (Urban et al., 2020).

Consistent with the 2015 sampling in the larger TBI cohort (*n* = 22), *Akkermansia* spp*., Anaerotruncus colihominis, Bacteroides* spp*., Clostridium* spp*., Collinsella* spp*., Faecalibacterium prausnitzii, Lactobacillus* spp*., Parabacteroides* spp*., Prevotella* spp*., Streptococcus* spp., and *Sutterella* spp. were detected in all five resamples (Table 2; Urban et al., 2020). Although not previously reported *Alistipes putredinis, Bifidobacterium* spp.*, Blautia* spp.*, Dialister* spp.*, Lachnoclostridium* spp., and *Veillonella* spp. were also universally detected and found to be abundant community members in the TBI profile (Table 2). Among the *Bacteroides* genus, *Bacteroides uniformis* was the most abundant species. *Bacteroides ovatus, Bacteroides stercoris*, and *Bacteroides vulgatus* were detected almost as frequently, consistent with the 2015 analyses (Urban et al., 2020). Notable, but not significant changes, in the average profile between 2015 and 2020 included reduction in abundance and detection of *Bacteroides caccae* (13/22 detection and 4E4 genomes in 2015 versus 2/5 detections and 5E2 genomes in 2020), *Bacteroides dorei* (13/22 and 5E4 versus 2/5 and 34 genomes), *B. vulgatus* (10/22 and 1.6E5 versus 3/5 and 7E3 genomes), *Lactobacillus fermentum* (17/22 and 2E3 versus 1/5 and 1 genome) and *Parabacteroides merdae* (18/22 and 4.9E4 versus 3/5 and 6E2 genomes; multi paired *t*-test, *p* = 0.05). Finally, *Lactobacillus reuteri* and *Lactobacillus salivarius*, were not detected in any of the five resamples consistent with the 2015 individual profiles.

**TABLE 2:** Metagenomic comparisons between TBI and control subjects.

Target	TBI			Control			TBI FD	BKY *p*-value	W *p*-value	FE *p*-value	Match 2015 FC
**Genus and species**	**Count**	**Avg**	**SD**	**Count**	**Avg**	**SD**					
*Akkermansia* spp.	5	2.7E+05	5.1E+05	3	6.7E+04	1.1E+05	4.01	0.36	0.34	0.44	Y
*Alistipes* spp.	5	6.9E+05	1.3E+06	5	1.1E+05	1.9E+05	6.32	0.36	0.39	1	NR
*Alistipes onderdonkii*	4	7.8E+05	1.5E+06	5	9.1E+04	1.9E+05	8.56	0.35	0.34	1	Y
*Alistipes putredinis*	5	6.3E+04	9.2E+04	5	1.5E+04	3.2E+04	4.15	0.36	0.34	1	NR
*Anaerotruncus colihominis*	5	2.3E+03	4.6E+03	5	1.3E+02	1.3E+02	17.98	0.12	0.10	1	Y
*Bacteroides* spp.	5	1.7E+05	3.2E+05	5	2.7E+05	2.2E+05	0.64	0.73	0.60	1	NR
*Bacteroides caccae*	2	5.4E+02	7.6E+02	4	1.2E+04	9.9E+03	0.04	0.23	0.08	0.52	Y
*Bacteroides cellulosilyticus*	1	3.4E+03	N/A	2	4.3E−03	4.9E−03	N/A	N/A	N/A	1	NT
*Bacteroides dorei*	2	3.4E+01	4.8E+01	4	2.2E+02	2.3E+02	0.15	0.15	0.15	0.52	N (1.03)
*Bacteroides fragilis*	2	3.1E+03	3.2E+03	0	N/A	N/A	N/A	N/A	N/A	0.44	NR
*Bacteroides massiliensis*	1	1.5E+01	N/A	2	1.2E+02	5.3E+01	0.12	N/A	N/A	1	Y
*Bacteroides ovatus*	3	1.0E+04	1.5E+04	5	4.1E+03	4.6E+03	2.48	0.18	0.13	0.17	NR
*Bacteroides plebeius*	2	1.8E+01	2.6E+01	0	N/A	N/A	N/A	N/A	N/A	0.44	NA
*Bacteroides sartorii*	4	4.2E+00	7.3E+00	2	4.3E+00	2.5E+00	0.97	0.23	0.22	0.52	N (11)
*Bacteroides stercoris*	3	1.2E+03	9.4E+02	4	1.1E+04	1.2E+04	0.11	0.15	0.15	1	N (1.56)
*Bacteroides stercorirosoris*	3	3.4E+02	5.2E+02	2	2.6E+01	3.6E+01	12.96	0.39	0.38	1	Y
*Bacteroides thetaiotaomicron*	2	1.8E+04	2.5E+04	4	4.1E+03	3.6E+03	4.42	0.11	0.11	0.52	Y
*Bacteroides uniformis*	4	1.4E+05	2.6E+05	5	1.2E+05	9.4E+04	1.2	0.13	0.05	1	Y
*Bacteroides vulgatus*	3	7.2E+03	8.9E+03	5	2.1E+04	2.1E+04	0.34	0.15	0.07	0.17	N (1.25)
*Bifidobacterium* spp.	5	1.3E+04	2.2E+04	5	3.5E+03	2.7E+03	3.82	0.41	0.37	1	NR
*Bifidobacterium bifidum*	2	2.2E+04	1.3E+04	2	2.7E+02	3.8E+02	79.21	0.44	0.41	1	N (0.39)
*Bifidobacterium longum*	3	7.7E+03	1.2E+04	4	1.9E+03	1.5E+03	3.98	0.21	0.09	1	NR
*Blautia* spp.	5	2.4E+06	3.2E+06	5	3.4E+06	3.1E+06	0.72	0.03	0.07	1	NR
*Clostridium* spp.	5	4.2E+02	2.6E+02	5	1.1E+03	2.1E+03	0.37	0.45	0.49	1	NR
*Clostridium leptum*	5	3.2E+02	1.9E+02	5	1.1E+03	2.1E+03	0.29	0.32	0.30	1	N (5.5)
*Clostridium symbiosum*	4	1.3E+02	1.1E+02	5	2.4E+01	2.5E+01	5.14	0.11	0.10	1	Y
*Collinsella* spp.	5	8.1E+04	1.5E+05	5	2.3E+05	1.6E+05	0.35	0.06	0.03	1	Y
*Corynebacterium* spp.	5	7.6E+05	4.0E+05	5	7.1E+05	1.1E+06	1.07	0.64	0.92	1	NR
*Corynebacterium propinquum*	2	3.6E+00	3.3E+00	0	N/A	N/A	N/A	N/A	N/A	0.44	NR
*Desulfovibrio* spp.	4	3.6E+03	1.2E+03	5	1.6E+04	2.4E+04	0.22	0.22	0.21	1	N (3.44)
*Dialister* spp.	5	1.9E+04	2.2E+04	5	7.7E+03	6.4E+03	2.45	0.05	0.06	1	NR
*Eubacterium* spp.	4	1.6E+05	1.7E+05	5	1.2E+05	1.1E+05	1.36	0.71	0.66	1	NR
*Eubacterium rectale*	4	1.9E+05	1.7E+05	5	1.2E+05	1.1E+05	1.67	0.17	0.08	1	NR
*Eubacterium siraeum*	4	5.7E+03	7.3E+03	3	2.5E+03	4.3E+03	2.28	0.39	0.37	1	NR
*Faecalibacterium prausnitzii*	5	2.9E+05	4.6E+05	5	5.3E+05	2.8E+05	0.55	0.01	0.01	1	Y
*Lachnoclostridium* spp.	5	2.0E+06	1.4E+06	5	2.2E+06	1.2E+06	0.93	0.05	0.02	1	NR
*Lactobacillus* spp.	5	7.0E+03	1.5E+04	5	1.3E+03	1.1E+03	5.54	0.44	0.44	1	NR
*Lactobacillus fermentum*	1	1.1E+00	N/A	5	4.4E+02	9.7E+02	0.002	N/A	N/A	0.048	N (15.6)
*Lactobacillus reuteri*	0	N/A	N/A	0	N/A	N/A	N/A	N/A	N/A	1	NA
*Lactobacillus rhamnosus*	5	2.1E+01	3.2E+01	2	3.8E+00	5.2E+00	5.42	0.39	0.37	0.17	NR
*Lactobacillus salivarius*	0	N/A	N/A	0	N/A	N/A	N/A	N/A	N/A	1	NA
*Megasphaera massiliensis*	0	N/A	N/A	2	3.0E+03	4.0E+03	N/A	N/A	N/A	0.44	NA
*Odoribacter* spp.	4	1.5E+04	1.6E+04	4	9.7E+03	4.9E+03	1.59	0.03	0.05	1	Y
*Parabacteroides* spp.	5	5.5E+03	8.3E+03	5	9.5E+03	5.2E+03	0.59	0.76	0.41	1	NR
*Parabacteroides distasonis*	3	1.9E+03	2.4E+03	5	1.8E+03	3.2E+03	1.11	0.30	0.28	0.17	Y
*Parabacteroides goldsteinii*	1	2.0E+04	N/A	5	3.3E+02	5.0E+02	60.32	N/A	N/A	0.048	NR
*Parabacteroides merdae*	3	6.0E+02	1.0E+03	5	7.4E+03	6.1E+03	0.08	0.13	0.05	0.17	Y
*Prevotella* spp.	5	1.7E+05	3.8E+05	5	2.2E+06	3.3E+06	0.08	0.24	0.24	1	Y
*Prevotella copri*	1	1.5E+04	N/A	3	6.0E+04	1.0E+05	0.25	N/A	N/A	0.52	Y
*Prevotella stercorea*	0	N/A	N/A	1	5.4E+02	N/A	N/A	N/A	N/A	1	NA
*Propionibacterium acnes*	1	1.1E+01	N/A	2	9.6E−01	1.1E−01	11.14	N/A	N/A	1	NR
*Ralstonia pickettii*	4	2.9E+03	3.2E+03	1	N/A	N/A	N/A	N/A	N/A	0.048	NR
*Ruminococcus* spp.	4	1.51E+04	1.21E+04	5	5.5E+04	6.2E+04	0.27	0.27	0.22	1	NR
*Ruminococcus bromii*	2	2.3E+03	1.3E+03	4	3.4E+03	5.5E+03	0.68	0.31	0.29	0.52	Y
*Streptococcus* spp.	5	1.1E+04	1.4E+04	5	3.1E+04	4.4E+04	0.34	0.19	0.19	1	Y
*Streptococcus salivarius*	2	1.4E+04	1.8E+04	4	3.3E+02	3.1E+02	42.29	0.16	0.14	0.52	Y
*Subdoligranulum* spp.	4	2.9E+04	3.1E+04	4	1.3E+04	9.3E+03	2.27	0.07	0.08	1	NR
*Sutterella* spp.	5	8.6E+04	1.5E+05	5	4.3E+04	3.1E+04	1.99	0.07	0.03	1	N (0.37)
*Veillonella* spp.	5	2.2E+02	2.8E+02	5	9.8E+02	1.8E+03	0.23	0.35	0.28	1	NR

Results for the TBI cohort are displayed in columns 2, 3 and 4. The Control results are shown in columns 5, 6 and 7. The count column shows the number of detections for each target out of the five samples in each cohort. The average genomic abundance (avg column; by qPCR) and standard deviation (SD column) for each cohort is shown. The fold difference (FD) between abundance of specified qPCR targets in the average TBI FMB compared to control FMBs is shown. Because of the complexity of these comparisons, several methods of statistical comparisons were performed as depicted. BKY, paired *t*-test with Benjamini, Krieger, and Yekutieli correction for false discovery; W, Mann–Whitney test; FE, Fischer’s exact test for detection differences. The results for the 2020 comparison were compared to those of the 2015 comparison with the original control group to determine if the fold change (FC) was not previously reported (NR) or matched [yes (Y) or no (N)] the prior data, discordances are shown as a numerical value.

Resample of the 2015 control cohort was not possible, so a new cohort of control fecal samples were analyzed similarly. The absolute abundance for each qPCR array target was normalized to total 16S rDNA copies and then compared to the average TBI FMB by paired and unpaired multiple *t*-test analyses and by Fisher’s exact test for binary comparisons (Table 2). These comparisons identified an additional 22 microbiota of interest that were either new to testing or not highlighted in 2015 (Urban et al., 2020). Across the previously reported targets, fold change differences (TBI versus control) for the 2015 cohorts were matched to those of the 2020 comparison to identify bacteria most likely involved in dysbiosis. With greatly reduced group sizes, variation was anticipated when TBI resamples were compared to the new non-TBI controls. Remarkably, only nine consistently tested targets showed a reversed average fold difference (Table 2) and helped refine the list of targets most likely associated with the altered metagenomic TBI profile. In fact, this approach revealed 19 genomic targets that matched the average fold difference reported for the 2015 sampling despite potential biological noise introduced by the new controls.

Considering the limited number of available subjects for resampling we also analyzed the statistical differences in detection between groups. This comparison found that *L. fermentum* and *Parabacteroides goldsteinii* were detected significantly more often in controls (5/5 controls but only 1/5 TBI; *p* = 0.048, Fisher’s exact test). *Ralstonia pickettii* was more commonly detected in TBI samples (5/5 TBI but only 1 control; *p* = 0.048). Differences in absolute abundance also were identified for those organisms detected in at least three samples from both cohorts by paired and unpaired comparisons. Five bacteria were found to have at least 1 *p*-value ≤ 0.05 and be higher in average abundance in control samples including *Blautia* spp. (1.4-fold, *p* = 0.03), *Collinsella* spp. (3-fold, *p* = 0.03), *F. prausnitzii* (1.8-fold, *p* = 0.01), *Lachnoclostridium* spp. (1.1-fold, *p* = 0.05), and *P. merdae* (12.3-fold, *p* = 0.05). Four bacteria were identified as more abundant in TBI by the same metrics including *B. uniformis* (1.2-fold, *p* = 0.05), *Dialister* spp. (2.5-fold, *p* = 0.05), *Odoribacter* spp. (1.6-fold, *p* = 0.05), and *Sutterella* spp. (2-fold, *p* = 0.03). Of these nine organisms, *B. caccae*, *B. uniformis*, *Blautia* spp., *Collinsella* spp., *Dialister* spp., and *Odoribacter* spp. had a consistent abundance difference in the 2015 and 2020 comparisons.

### TBI FMB metatranscriptome analyses revealed distinct community structures for bacteria that contributed RNA

The metagenomic analyses indicated a stable TBI FMB community profile that was substantially different from 2 distinct control cohorts sampled 5 years apart. The differences observed between TBI and control FMB metagenomic profiles failed to identify functional changes associated with the distinct communities. To address the consequence of the distinct profile, metatranscriptomics (metaTx) were completed on the five TBI fecal resamples that were compared to the 2020 controls. In addition to providing the opportunity to compare bacterial behavior in the TBI and healthy GI environments, metaTx identified the fecal bacteria that contributed RNA. This analysis distinguished viable, metabolically active bacteria from the bacteria that were detected metagenomically but were not associated with active transcription. Using 75 bp, paired-end reads we mapped sequences to genus and species levels supporting analyses of community structures, pathway abundance and ultimately the identification of bacteria most contributory to the differences and functional consequences of the shifted TBI FMB profile.

As an initial analysis of the metaTx, the list of bacterial genera that contributed the highest numbers of sequence reads in the average TBI and control community structure were compiled considering only those that were detected in ≥2/5 samples in each cohort. As shown in Table 3, based on the percent of total mapped RNA reads, there were 8 common genera in the top 10 contributors between the TBI and control metaTx averages including *Bacteroides*, *Faecalibacterium, Alistipes, Propionibacterium, Ralstonia, Eubacterium, Alcaligenes*, and *Subdoligranulum*. Others have reported similar findings for microbial contributors to the core FMB metatranscriptome (Abu-Ali et al., 2018). The other 2 exclusive genera in the top 10 contributors for the average TBI profile were *Corynebacterium* and *Pseudomonas* while *Parabacteroides* and *Ruminococcus* were the distinct members of the top 10 contributors in the control profile. In the average TBI metaTx profile, the *Corynebacterium* genus contributed the most RNA but was 12th in contribution for controls (9% versus 2.7% of total RNA sequences in TBI versus controls; Table 3). The most robust contribution in the genus was mapped to *Corynebacterium kroppenstedtii* (average of 4.1% for TBI and present in all 5 samples) which was also the most abundant contributor in controls for this genus (2.1%, present in 4/5 samples). Despite the difference in RNA contribution, the *Corynebacterium* genus was present in every sample and statistically indistinguishable in control and TBI FMBs (Table 2). The second largest RNA contribution to the TBI metaTx profile was attributed to the *Alistipes* genus (7.2% versus 4.6%; Table 3). Metagenomically, the *Alistipes* genus was detected in all samples in both cohorts but was 6.3-fold more abundant in the average TBI community (Table 2). Among the *Alistipes* species, *Alistipes onderdonkii* was the most contributory (3.26% of total RNA) but was only detected in 3/5 TBI samples. *A. putredinis* was detected in 4/5 TBI samples and was responsible for 2.4% of the RNA detected in the average metaTx profile.

**TABLE 3:** Relative RNA contribution of the top 10 genera for TBI and non-TBI FMB profiles.

Contributing genus TBI FMB	Percent of total RNA contribution		Contributing genus Control FMB
***Corynebacterium***	9.02	8.32	***Parabacteroides***
*Alistipes*	7.22	6.67	*Bacteroides*
*Eubacterium*	6.99	6.45	***Ruminococcus***
*Subdoligranulum*	6.65	5.77	*Faecalibacterium*
*Bacteroides*	6.5	4.59	*Alistipes*
*Propionibacterium*	6.39	4.29	*Propionibacterium*
*Ralstonia*	5.82	4.21	*Ralstonia*
***Pseudomonas***	5.48	3.87	*Eubacterium*
*Alcaligenes*	5.19	3.67	*Alcaligenes*
*Faecalibacterium*	4.58	3.29	*Subdoligranulum*

Genera shown in bold text are unique to the top 10 contributors of the indicated cohort. Percentage of total RNA contribution values are based on normalized counts of assigned RNA sequences.

In the controls, the highest percentage of RNA sequences were attributed to *Parabacteroides* (8.3% versus only 2.3% in TBI making it 13th most contributory); *Bacteroides* was the second most contributory genus (6.7%). From the metagenomic perspective, *Parabacteroides* genomes were detected in every control and TBI sample at similar abundance suggesting that the TBI environment altered the transcriptional behavior of *Parabacteroides* or that the significantly more frequently detected *P. goldsteinii* was a main contributor to the RNA differences. Similarly, *Bacteroides* spp. genomes were detected in every TBI and control sample with a slightly higher abundance in controls (Table 2). However, the *Bacteroides* member species genomic presence and abundance were distinctive (Table 2). *Ruminococcus* was the third most contributory genus in the controls (11th in TBI) although only 4/5 samples were positive for genomes and there was a 3.7-fold lower genomic abundance in controls than TBI (Table 2).

### MetaTx data processing identified FMB molecular pathways that may contribute to long-term sequelae

Individual metaTx profiles were bioinformatically processed to identify a cohort average number of expressed bacterial genes (ASaiM) and comparisons to identify differently expressed genes (DEGs, *p* < 0.05, *t*-test). On average, TBI samples contained 1.57E4 expressed genes (range of 1.23E4–2.22E4) while controls averaged 9.79E3 expressed genes (range of 4.95E3–1.59E4). Consistent with prior reports (Abu-Ali et al., 2018), we observed a “core metaTx” that had consistent detection and abundance between the two cohorts allowing those genes to be eliminated from further consideration. Comparison of the average profiles revealed nearly 700 DEG between the average TBI and control profile contributed by over 100 bacteria. The metaTx profiles were subjected to ASaiM workflows (Batut et al., 2018; Mehta et al., 2021) to identify molecular pathways that were differently expressed between the cohorts. The average TBI profile had 311 detected pathways with 264 in the average control profile. The most abundantly expressed pathway for both profiles was glycolysis IV (14.8% and 2.96% of the total for control and TBI, respectively; Table 4). There were 7 additional common pathways in the top 10 for both cohorts. The pathways for coenzyme A biosynthesis II and 5 aminoimidazole ribonucleotide biosynthesis I were unique to the TBI top 10 (1.7% and 1.5% of total, respectively; Table 4). Glycolysis III and VI pathways were distinct to the top 10 in the control profile (4.4% and 1.9% of total, respectively).

**TABLE 4:** Top 10 most abundantly expressed FMB metabolic pathways in each cohort.

Metabolic pathway TBI FMB	Percent of total		Metabolic pathway Control FMB
Glycolysis IV	2.96	14.8	Glycolysis IV
Adenosine ribonucleotides *de novo* biosynthesis	2.86	5.04	Adenosine ribonucleotides *de novo* biosynthesis
L valine biosynthesis	2.63	4.44	**Glycolysis III**
Pyruvate fermentation to isobutanol	2.45	3.13	L valine biosynthesis
Guanosine ribonucleotides *de novo* biosynthesis	2.23	2.83	Guanosine ribonucleotides *de novo* biosynthesis
5 aminoimidazole ribonucleotide biosynthesis II	2.04	2.69	Pyruvate fermentation to isobutanol
Superpathway of 5 aminoimidazole ribonucleotide biosynthesis	2.04	2.08	5 aminoimidazole ribonucleotide biosynthesis II
**Coenzyme A biosynthesis II**	1.73	2.08	Superpathway of 5 aminoimidazole ribonucleotide biosynthesis
L isoleucine biosynthesis I	1.71	1.93	**Glycolysis VI**
**5 aminoimidazole ribonucleotide biosynthesis I**	1.57	1.91	L isoleucine biosynthesis I

The average relative abundance (% of total mapped pathways) are shown for the 10 most abundant pathways in each cohort. Pathways unique to the top 10 of each cohort are shown in bold. Pathway labels are assigned by the described workflow and are matched to the MetaCyc database.

**FIGURE 2:**
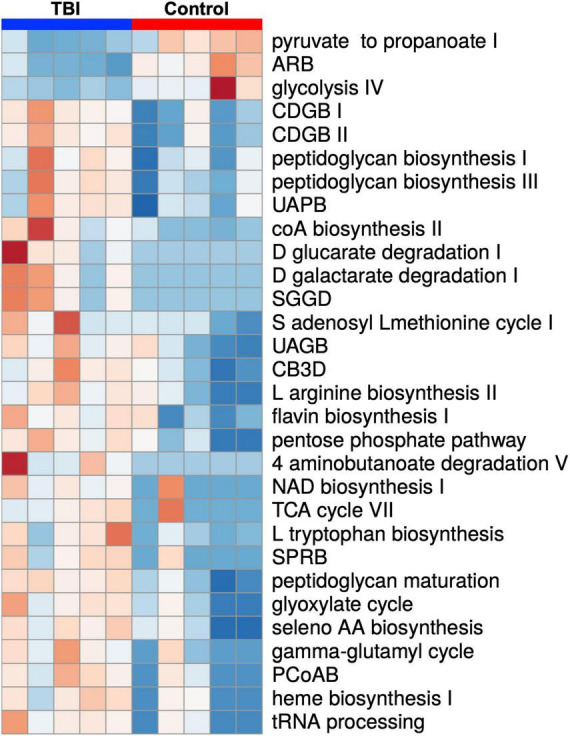
A heat map depiction of the 30 significantly differently expressed biochemical pathways identified by comparison of the average TBI and control metatranscriptomic data. Individual expression levels for each of the FMB profiles is shown (red is high expression and blue is low expression). TBI samples are clustered on the left (blue) and controls on the right (red). Three pathways were significantly more abundant in controls (top 3 labels on the right) and 27 were more abundant in the TBI (*p* < 0.05 multiple *t*-test). Additional detail for specific values can be found in Figure 3. Pathway names are assigned by the ASAIM workflow and match the MetaCyc database. ARB, adenosine ribonucleotides *de novo* biosynthesis; CDGB, CDP diacylglycerol biosynthesis pathway I or II; UAPB, UDP N acetylmuramoyl pentapeptide biosynthesis; SGGD, superpathway of D glucarate and galactarate degradation; UAGB, UDP N acetyl D glucosamine biosynthesis I; CB3D, chorismate biosynthesis from 3 dehydroquinate; SPRB, superpathway of pyrimidine ribonucleotides *de novo* biosynthesis; PCoAB, pantothenate and coenzyme A biosynthesis I.

**FIGURE 3:**
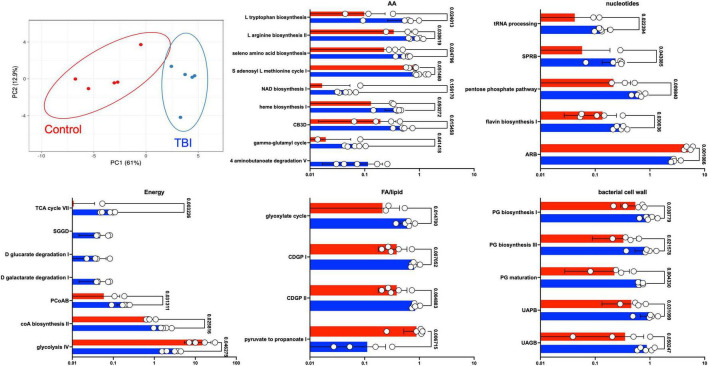
Differently expressed pathways grouped by core metabolic process. The top left panel depicts a PCA of the distinctions based on the expression pattern of the 30 pathways between TBI (blue) and control (red) FMB metatranscriptome data. The other five panels are scatterplots illustrating the average expression level of each pathway as a percent of total mapped RNA sequences with circles denoting individual profile values. SEM values are shown as whiskers and *p*-values are shown (unpaired Welch’s *t*-test with *p* < 0.05 considered significant). Pathway abbreviations are as shown in the legend to Figure 2.

Statistical comparison of normalized pathway abundance led to the identification of 30 bacterial pathways that were either significantly different in abundance (*p* < 0.05, unpaired *t*-test) or were detected in ≥80% of the samples in only one of the cohorts (binary distinction; Figure 2). This list included pathways that grouped into categories for energy utilization, AA metabolism, fatty acid and lipid metabolism, nucleotide metabolism and bacterial cell wall component production. Unsupervised clustering of individual samples, considering only these 30 pathways, illustrated robust distinctions between the cohorts (Figures 2, 3). Of the 30, 27 were more abundant or more often detected in the average TBI profile while the remaining 3 were significantly more abundant in controls.

To address a main goal for this study, bacteria that were most contributory to the differences between the profiles (≥ 1% RNA contribution) were identified by mapping DEG sequences associated with the 30 pathways to genus and species. At the genus level, *Bacteroides* and *Faecalibacterium* were robust contributors with 62% of the DEG for these pathways in the control profiles mapped to these 2 genera (52% and 10%, respectively; Table 5). In TBI, these genera contributed 6% and 7%, respectively. Interestingly, the largest contributions in the TBI samples were mapped to *Ralstonia* (11%) and *Corynebacterium* (10%) genera. At the species level, *B. uniformis* and *B. vulgatus* were responsible for the greatest differences in contributions to these pathways (10% and 22%, respectively in controls versus 2% each for TBI; Table 5). *Faecalibacterium* sequences mapped to *F. prausnitzii* in both cohorts. *Ralstonia* sequences mapped to *R. pickettii*.

**TABLE 5:** Top bacterial RNA contributors to the 30 differently expressed pathways.

Genus and species	% contribution (>1% only)	
	TBI	Control
*Achromobacter piechaudii*	2	1
*Alcaligenes faecalis*	1	1
*Alistipes onderdonkii*	6	3
*Alistipes putredinis*	2	3
***Bacteroides* spp.**	**6**	**52**
*Bacteroides caccae*		3
*Bacteroides coprocola*		7
*Bacteroides massiliensis*		2
*Bacteroides ovatus*		2
*Bacteroides stercoris*		4
*Bacteroides uniformis*	2	10
*Bacteroides vulgatus*	2	22
*Bifidobacterium adolescentis*	5	
*Blautia* spp.	4	2
*Corynebacterium accolens*	3	
*Corynebacterium kroppenstedtii*	5	1
*Corynebacterium propinquum*	2	
*Enhydrobacter aerosaccus*	3	
*Eubacterium rectale*	7	3
*Faecalibacterium prausnitzii*	7	10
*Lachnospiraceae* 2_1_58FAA	2	
*Parabacteroides distasonis*	1	2
*Propionibacterium acnes*	5	1
*Pseudomonas aeruginosa*	3	2
***Ralstonia pickettii***	**11**	**4**
*Ruminococcus gnavus*	2	
*Ruminococcus obeum*	1	2
*Ruminococcus* sp_5_1_39BFAA	4	
*Serratia marcescens*	3	1
*Streptococcus* spp.	4	3

Bacterial genera or species that contribute to the 30 distinctive pathways identified by statistically significant differences in relative abundance or binary detection are shown. Bacteria were only included if their contribution values were >1% of the total for the pathways. Bolded bacteria and values indicate the greatest distinction in bacterial contribution between cohorts.

**FIGURE 4:**
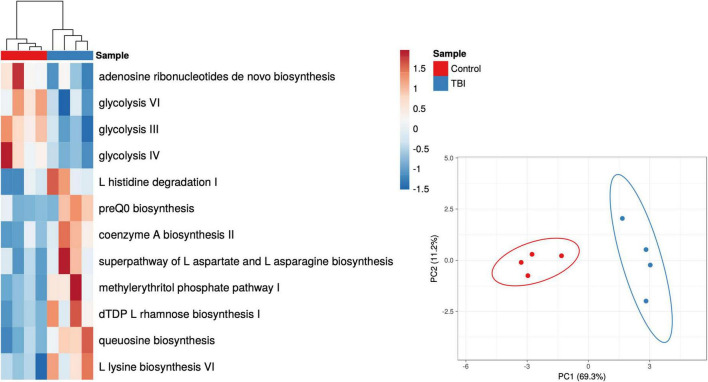
Heat map and PCA plots of the 12 differently expressed pathways based on *Bacteroides* contributions only. The individual *Bacteroides* pathway profiles for subjects with TBI (blue) and healthy controls (red) are depicted as a heat map representing the 12 that showed significant differences in abundance (*p* < 0.05 multiple *t*-test; red to blue scale bar shows percent of total). The PCA illustrates the distinction provided illustrating the complexity of the transcriptional adaptation of this genus to the TBI and health control GI environments.

Given the robust differences observed for the *Bacteroides* genus, we considered only those RNA sequences that mapped to this genus. Pathways mapped to *Bacteroides* in each individual sample revealed one sample in each cohort that had minimal contributions that were eliminated to avoid signal dilution. The remaining 4 samples in each cohort were statistically compared leading to the identification of 12 pathways that were significantly different in abundance between the 2 cohorts (Figure 4 and Table 6). Among the *Bacteroides*-specific differences, eight pathways were significantly more abundant in the average TBI profile and four were significantly higher in the control metaTx (Table 6). Three of the four were glycolysis pathways (III, IV, and VI). The fourth was the adenosine ribonucleotides *de novo* biosynthesis pathway. Glycolysis IV and the adenosine pathway were in the previously described top 30 and were higher in the control cohort profile (Figure 2). The reduced levels of these energy use pathways in the TBI metaTx profiles supports a distinct environment leading to use of alternate energy metabolism pathways. The eight pathways expressed more abundantly in the TBI *Bacteroides*-specific profile included novel AA and nucleotide metabolism pathways and a pathway associated with phosphoantigens that activate gamma delta T cells (Feurle et al., 2002; Table 6). The final pathway, coenzyme A biosynthesis II was noted in the top 30 and also involves metabolism of AAs.

**TABLE 6:** Differently expressed *Bacteroides*-specific pathways.

Pathway	Percentage of total		TBI/control	*p*
	TBI avg	Control avg		
Glycolysis IV	7.94	24.3	−3.1	0.02
dTDP L rhamnose biosynthesis I	4.32	1.69	2.6	0.03
Adenosine ribonucleotides *de novo* biosynthesis	4.23	6.58	−1.6	0.03
Glycolysis III	4.06	8.19	−2	0.001
L histidine degradation I	3.74	1.58	2.4	0.05
Glycolysis VI	2.12	4.34	−2	0.01
Queuosine biosynthesis	1.95	0.58	3.4	0.01
PreQ0 biosynthesis	1.83	0.437	4.2	0.05
Methylerythritol phosphate pathway I	1.8	0.541	3.3	0.03
L lysine biosynthesis VI	1.74	0.856	2	0.02
Coenzyme A biosynthesis II	1.64	0.212	7.7	0.02
Superpathway of L aspartate and L asparagine biosynthesis	0.915	0.229	4	0.05

The 12 significantly different pathways contributed by *Bacteroides* spp. are shown with the average % of total RNA depicted and the related fold change (FC) and *p*-value (multiple *t*-test).

## Discussion

Acute TBI has been shown to cause FMB dysbiosis in both clinical and animal model studies (Treangen et al., 2018; Nicholson et al., 2019; Brenner et al., 2020; Pathare et al., 2020; Opeyemi et al., 2021; Soriano et al., 2022). Based on studies of individuals with debilitating moderate to severe TBIs, we continue to test the hypothesis that the dysbiotic TBI FMB becomes a stabilized norm in some patients and may cause or contribute to long-term fatigue and altered cognition years after injury. Using multi-omic approaches, the stability of the dysbiotic FMB was shown in five individuals who provided a fecal sample 5 years after the initial study and, on average, 25 years after TBI. Each TBI FMB kinetic pair was found to be significantly similar supporting the conclusion that a stable dysbiotic relationship was formed between FMB community members and the TBI GI environment. Further, by comparing the metagenomic profiles to a new cohort of age- and gender-matched controls, FMB organisms common to the prior and current comparisons were identified, revealing several genera associated with TBI dysbiosis and refining prior outcomes (Urban et al., 2020). Ongoing work led to additional qPCR targets identified in the average TBI FMB community including *R. pickettii*. The genomic DNA for *R. pickettii* was detected in all five TBI FMB communities while only one control sample had detectable DNA suggesting a potential role in TBI-related dysbiosis and symptoms. By comparison to the new control cohort, *L. fermentum* and *P. goldsteinii* were found almost exclusively in controls. Finally, control FMBs had higher abundance of *Blautia* spp., *Collinsella* spp., *F. prausnitzii*, *Lachnoclostridium* spp., and *P. merdae* genomes consistent with prior observations (Urban et al., 2020) leading to the prediction that these organisms may provide metabolic functions associated with eubiosis that are reduced or lost in the dysbiotic TBI FMB.

Metagenomic WGS analyses predict encoded functions that may contribute to dysbiosis but fail to confirm that specific operons are expressed so metaTx comparisons were completed. These analyses identified both contributing bacteria and pathways enhancing understanding of the TBI FMB dysbiosis. This small, focused study revealed significant differences in multiple core metabolic functions and further refined the organisms most likely involved in the dysbiosis by mapping DEGs. Unexpectedly, these analyses illustrated that the TBI FMB had increased numbers of expressed genes and pathways suggesting enriched functional diversity and alternate pathway usage compared to FMBs from healthy subjects. Several genera (e.g., *Prevotella*) previously identified metagenomically (Urban et al., 2020) failed to show functional contributions while others (e.g., *Ralstonia*) proved distinctive in both genomic and transcriptomic outcomes. Mapping detected sequences revealed that *Parabacteroides* spp. contributed the most RNA to the control profile. However, significant DEGs were not mapped to this genus suggesting that this genus contributed only to common core metabolic processes. In contrast, the distinction in the *Bacteroides* genus was substantial and complicated. *Bacteroides* spp. contributed the second highest amount of RNA to the control cohort that was indistinguishable from the amount in TBI profiles (both ∼6.5%; Table 3). However, when considering only significant DEGs and pathways that mapped to *Bacteroides*, 52% mapped to *Bacteroides* spp. in the controls while only 6% mapped to this genus in TBI profiles (Table 5) supporting a strong eubiotic contribution consistent with other reports (Elfil et al., 2020; Dinan and Dinan, 2022).

Fecal microbiome bacteria produce a variety of metabolites that influence or directly signal neuronal and endocrine changes (Sarkar et al., 2018; Dinan and Dinan, 2022). The complex web of metabolite usage within and between community members leads to selection pressures that favor some bacteria while forcing others to seek alternate means for survival. The metaTx data established 5 broad differences represented by the 30 differently expressed pathways including major differences in glycolysis pathway expression (Figures 2, 3). Of the 30 pathways, the 3 that were significantly more abundant in the control profile (pyruvate fermentation to propanoate I, adenosine ribonucleotides *de novo* biosynthesis and glycolysis IV) revealed contributions missing from the TBI FMB.

Pyruvate fermentation to SCFAs and nucleotide levels (potentially contributed by commensal bacteria) have both been connected to improved outcomes for TBI recovery (Lusardi, 2009; Yanckello et al., 2022a,b; Zhao et al., 2023). Interestingly, as reviewed by Gomes et al. (2011), work on extracellular adenosine in a variety of brain disorders has led to recognition that adenosine receptor antagonists positively promote synaptic plasticity that may ultimately involve control of glutamatergic transmission or a variety of other brain homeostasis pathways. Remarkably, research on adenosine receptor antagonists, including caffeine and other methylxanthine compounds, led to the approval of istradefylline as a treatment for Parkinson’s disease (Berger et al., 2020). Reduced abundance of the adenosine pathway in the TBI profile and increase in pyrimidine synthesis related pathways (SPRB and flavin biosynthesis) suggests environmental differences leading to FMB adaptation. Finally, elevated expression of the pentose phosphate pathway also relates to carbon usage and homeostasis consistent with the more diverse metaTx requirements for the TBI FMB.

Substantial differences in the types of glycolysis-associated RNAs between cohorts may be central to the dysbiotic functions associated with the TBI FMB. Glycolysis is a central amphibolic metabolic process in bacteria contributing both energy (catabolic) and molecular precursors (anabolic) to other pathways in the form of pyruvate. Different types of bacterial glycolysis metabolize different sugars, ultimately cleaving glucose into as many as four ATP molecules and two pyruvates (Wolfe, 2015). Glycolysis (pathway IV) was a common pathway when comparing the metaTx profiles but alternative versions of glycolysis (glycolysis III and VI) were distinct to the top 10 most abundant pathways in the controls. This suggests that TBI FMB communities must be using alternate means of ATP generation and may ultimately contribute less pyruvate to the metabolome, limiting available precursors for other pathways. These differences could be produced by environmental activation of different operons or distinctly encoded pathways based on the bacterial genomes present. In support of distinctive sugar usage, three pathways that degraded glucarate or galactarate were exclusively observed in each of the individual TBI FMBs (Figure 3). Finally, increased abundance of the glyoxylate cycle RNAs in the TBI FMB suggest the need to create additional glucose in support of bacterial functions perhaps addressing predicted pyruvate deficiency.

Pyruvate is a precursor in many pathways, is utilized to synthesize AAs (e.g., alanine, valine, and leucine) and is converted to propionate by *Corynebacteria*, *Propionibacteria*, and *Bifidobacteria* (Wolfe, 2015). Other glycolysis metabolites are used to synthesize histidine and serine and drive production of lipids and vitamins. *Bacteroides* pathway analyses found reduced abundance of glycolysis in the TBI FMB as well as increased degradation of selected AAs (e.g., histidine) (Table 6). This increases the likelihood of reduced systemic availability of specific AAs based on both reduced synthesis and increased degradation for energy production that could contribute to the clinical hypoaminoacidemia observed following TBI (Børsheim et al., 2007; Dai et al., 2011; Durham et al., 2017). At the species level, *B. uniformis* and *B. vulgatus* represented the largest proportion of mapped *Bacteroides* RNAs in the controls, consistent with eubiotic functions (Wang et al., 2021; Dinan and Dinan, 2022). Genomic levels of *B. uniformis* were higher in the TBI FMB illustrating the limitation of metagenomics and exemplifying the complexity surrounding development of probiotics. Similarly, *B. vulgatus* is associated with ulcerative colitis (Mills et al., 2022) suggesting that the contextual influences of environment and community composition must be considered when attempting to assign probiotic status.

Among the AA metabolism differences (Figure 3), degradation pathways observed in the TBI profile utilized aspartate, glutamate, and glutathione (NAD biosynthesis, heme biosynthesis, and gamma-glutamyl cycle) as precursors supplying products for other pathways including AA synthesis and transport. In contrast, several biosynthesis pathways for specific AAs were more abundant in the TBI metaTx (tryptophan, arginine, and seleno AAs). The chorismate biosynthesis pathway (Figure 3, CB3D) can lead to synthesis of aromatic AAs including phenylalanine, tyrosine, and tryptophan consistent with the observed activation of the biosynthetic pathways. Finally, the 4 aminonbutanoate (GABA) degradation V pathway exclusively expressed in the TBI FMBs suggests a direct effect of the dysbiotic community on the availability of this major inhibitory neurotransmitter that will require additional study. The increased abundance of AA metabolism pathways in the TBI FMB and reduced abundance of pathways for pyruvate fermentation to produce lipids and SCFAs further illustrate adaptations that reflect responses to the TBI GI environment and/or the stable FMB selection.

The fifth group of more abundant pathways in the TBI FMB included bacterial cell wall component biosynthetic machinery for peptidoglycan and coenzyme A (Figure 3) that likely favors reproduction of pathobiont species. CoA serves as a cofactor for production of phospholipids, SCFAs and TCA cycle components illustrating the complexity of overlap associated with the adaptive transcription in FMB communities. Bacterial peptidoglycan in cell walls protects against environmental stress but also triggers inflammatory responses through interactions with pathogen recognition receptors. The role of inflammation remains unclear with some reports indicating it enhances neuroprotection while others show associated damage and post-TBI sequelae leading to controversial connections and inconclusive therapeutic strategies (Cederberg and Siesjö, 2010; Corps et al., 2015; Hanscom et al., 2021). Careful analysis of larger FMB metaTx datasets will be needed to confidently identify pathobionts adding inflammatory molecules to the systemic milieu.

**FIGURE 5:**
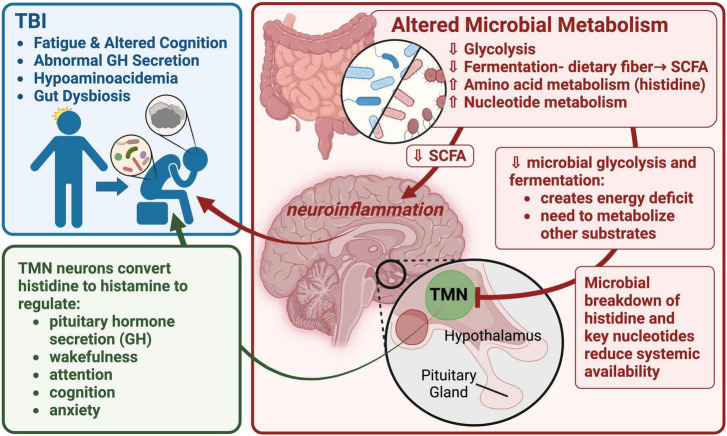
A graphic summary of the metaTx findings and potential neuroendocrine impacts. This image depicts a working model focused on altered TBI FMB metabolism of histidine, other AAs and nucleotides leading to impacts on histaminergic neuron function in the tuberomammillary nucleus (TMN) of the hypothalamus. Reduced production of histamine in the TMN would have a cascade effect including dysregulation of pituitary functions including growth hormone (GH) secretion. Created with BioRender.com.

As part of a working model of FMB contribution to long-term TBI sequelae, we hypothesize that the observed increase in histidine degradation (significantly more abundant in *Bacteroides*-specific TBI FMB pathways; Table 6), and distinct degradation and synthesis of other AAs as well as increased gamma-glutamyl cycle pathway expression support a role for AA metabolism in fatigue and cognitive issues following TBI (graphically summarized in Figure 5). A reduction in available histidine would be predicted to reduce conversion to histamine, a potent neurotransmitter. Histidine is converted to histamine by histaminergic neurons in the tuberomammillary nucleus located in the hypothalamus that serves a hub to link key brain regions associated with arousal, cognition, sleep, and GH secretion (Haas and Panula, 2003; Haas et al., 2008). Individuals with a history of TBI and BIAFAC symptoms had reduced max GH values (<10 ng/ml) following a glucagon stimulation test (Mossberg et al., 2017; Wright et al., 2020; Yuen et al., 2020) and hypoaminoacidemia following a standardized meal (Durham et al., 2017). GH replacement therapy improved symptoms (Mossberg et al., 2017; Wright et al., 2020; Yuen et al., 2020). In animal model studies, dietary and systemically administered histidine substantially increased plasma GH levels via pathways dependent on functional conversion to histamine by histidine decarboxylase (Maeda and Frohman, 1978; Cacabelos, 1991; Bruno et al., 1993). There has been almost a decade of connection between the histidine/histamine system and nervous system dysfunction including associations with autism spectrum disorder, Tourette’s syndrome, and other neuropathologies (Wright et al., 2017; Moro et al., 2020). Very recently, elegant mapping of histaminergic neuron fibers revealed a heterogenic projection pattern that reached a large number of brain regions with little overlap (Lin et al., 2023) suggesting histidine deficiency could have substantial impacts. Disruption of the histaminergic system has been associated with dysregulation of sleep-wake cycle (fatigue) and cognitive functions (Passani et al., 2000; Tashiro et al., 2002; Alvarez, 2009; Yoshikawa et al., 2021). Collectively these data support additional testing of the hypothesis that TBI FMB dysbiosis creates chronic histidine deficiency leading to dysregulated release of GH ultimately contributing to the observed profound fatigue and cognition issues in subjects with BIAFAC (Figure 5).

Histidine degradation and other AA metabolism pathways were mapped back to a select set of bacteria associated with greater contributions in TBI FMBs including *R. pickettii*. A member of the Burkholderiales order, *R. pickettii* was originally isolated from moist soil and water samples. It has since been reported as a FMB community member that impacts host functions (Udayappan et al., 2017; Kim et al., 2021) but has not been reported in the context of TBI. It is possible that the observed DEGs in our study were incorrectly mapped based on the 75 bp reads so additional studies will be required to confirm the functional role for *R. pickettii* and the other bacteria to establish if their transcriptional contributions are causally associated with TBI sequelae including abnormal GH and hypoaminoacidemia.

Among the limitations of this focused characterization study was the small cohort of subjects who provided a follow up fecal sample. Despite the small cohort, we confirmed lasting stable distinctions in the TBI FMB metagenome with strong statistically significant functional impacts identifying several metabolic consequences related to neuroendocrine dysfunction. Larger studies are warranted with focused analyses of the identified differences in core metabolic processes as well as functional reconstruction studies in *ex vivo* and germ-free mouse model systems. The RNA sequence mapping also must be confirmed by alternate methods to address inaccuracies cause by conservation of gene sequences that complicate accurate assignment to species levels. Associating multiple DEGs with differently expressed pathways reduces such errors and increases confidence in this screening method that supports development of testable hypotheses and directing next steps.

Translationally, completion of these studies will not only help to elucidate the impacts of the dysbiotic TBI FMB but should identify novel targets for prebiotic, probiotic, and molecular approaches targeted to the bacteria in the GI tract. There are a number of studies that tested general probiotics or FMB transplants in the context of TBI or other neurotrauma that showed mixed results (Kigerl et al., 2016; Brenner et al., 2017; Rice et al., 2019; Yi et al., 2019; Yanckello et al., 2022a,b) supporting a need for greater specificity in targeting. Based on the observed SCFA metabolism differences in the TBI FMB, we recently completed a trial of a prebiotic fructooligosaccharide dietary supplement in subjects who have moderate to severe TBI (NCT04949607). Such prebiotics create SCFAs through fermentation by several bacterial genera including some identified in this study. Data are being analyzed but the supplement was tolerated and appears to have had positive impacts on fatigue and cognitive measures (unpublished findings). Based on the current data implicating multiple metabolic processes, larger cohorts, and additional metaTx analyses will be needed to select optimal combinations of prebiotics, probiotics, and targeted molecules for optimal designs for clinical testing.

Interestingly, treatment of medically refractory epilepsy has included implementation of a ketogenic diet since the 1920s (Peterman, 1924) suggesting the reported dysbiosis associated with epilepsy may also reflect alternate energy metabolism and even distinct amino acid utilization as expertly reviewed by several groups (Holmes et al., 2020; Lum et al., 2020; Chatzikonstantinou et al., 2021). The published studies in clinical cohorts and in animal models primarily employed metagenomic analyses and were, like this report, limited by relatively small cohorts but illustrated some common findings that need to be further studied. In particular, several indicated reductions in the relative abundance of *Bacteroides* spp. (Lum et al., 2020; Chatzikonstantinou et al., 2021) and functional predictions of impacts on the acetyl-CoA pathway and subsequent potential for degradation of essential amino acids and consequential changes in GABA and other neurotransmitters. The intriguing similarities as well as connections to other neuropathologies support completion of addition studies including the use of metatranscriptomics to better address functional profile differences.

## Conclusion

Long-term sequelae related to TBI are relatively common with nearly 30% of survey participants reporting fatigue and cognitive symptoms within 6 months of a TBI and over 15% still experiencing symptoms 5 years later (Wright et al., in review, *PLoS One*). Others have reported that most patients with a TBI have lingering symptoms that slowly improve over the 5 years following injury (Nelson et al., 2023). Such data strongly support a TBI initiated cascade that may create a stable dysbiotic FMB impact. Working to address the stability of the dysbiotic TBI FMB we had the benefit of five subjects who were willing to provide a fecal sample 5 years after our original analysis which confirmed that dysbiosis associated with TBI decades earlier remained stable. By comparison to two temporal and geographically disparate control cohorts, we refined the list of organisms that define TBI FMB dysbiosis and identified potential prebiotic/probiotic intervention opportunities that will need to be carefully studied as effectively reviewed in the past (Brenner et al., 2017). Through metaTx analyses, functional impacts consistent with clinical deficiencies in essential AAs, SCFAs and altered energy utilization were confirmed. Although the gut-brain axis involves well established correlations, causative mechanisms are not understood. Using an established model of FMB dysbiosis and neurologic symptoms, we identified metabolic pathways expressed in the FMB that affect bioavailability of key signaling molecules, essential nutrients, and neurotransmitter precursors. These findings help initiate directed studies to better understand the underlying issues and the potential for interventions to address the long-term consequences of TBI on the gut-brain axis in some patients.

## Data availability statement

The datasets presented in this study can be found in online repositories. The names of the repository/repositories and accession number(s) can be found below: https://www.ncbi.nlm.nih.gov/, PRJNA1005267.

## Ethics statement

The studies involving humans were approved by the Advarra IRB (Pro00027812) for subjects at the CNS facility (Bakersfield, CA) or by the University of Texas Medical Branch (UTMB) IRB for control samples collected in Galveston County, TX. The studies were conducted in accordance with the local legislation and institutional requirements. The participants provided their written informed consent to participate in this study.

## Author contributions

RP: Conceptualization, Data curation, Formal analysis, Funding acquisition, Investigation, Methodology, Project administration, Supervision, Writing – original draft, Writing – review & editing. AM: Data curation, Formal analysis, Investigation, Methodology, Writing – review & editing. RU: Conceptualization, Funding acquisition, Investigation, Project administration, Writing – review & editing. MS-M: Conceptualization, Investigation, Project administration, Supervision, Writing – review & editing. TW: Data curation, Formal analysis, Writing – review & editing. CM: Data curation, Investigation, Methodology, Writing – review & editing. KMR: Investigation, Project administration, Supervision, Validation, Writing – review & editing. CD: Data curation, Investigation, Project administration, Writing – review & editing. KM: Investigation, Methodology, Writing – review & editing. JV: Formal analysis, Investigation, Methodology, Writing – review & editing. PA: Formal analysis, Investigation, Methodology, Writing – review & editing. LK: Investigation, Methodology, Project administration, Writing – review & editing. GC: Data curation, Investigation, Methodology, Writing – review & editing. KR: Investigation, Project administration, Validation, Writing – review & editing. MM: Investigation, Methodology, Writing – review & editing. BM: Investigation, Methodology, Project administration, Resources, Writing – review & editing, Conceptualization, Funding acquisition.

## Footnotes

1
https://usegalaxy.eu/

2
https://www.socscistatistics.com/tests/fisher/default2.aspx

## References

[B1] Abu-Ali G. S. Mehta R. S. Lloyd-Price J. Mallick H. Branck T. Ivey K. L. (2018). Metatranscriptome of human faecal microbial communities in a cohort of adult men. *Nat. Microbiol.* 3 356–366. 10.1038/s41564-017-0084-4 29335555 PMC6557121

[B2] Abubucker S. Segata N. Goll J. Schubert A. M. Izard J. Cantarel B. L. (2012). Metabolic reconstruction for metagenomic data and its application to the human microbiome. *PLoS Comput. Biol.* 8:e1002358. 10.1371/journal.pcbi.1002358 22719234 PMC3374609

[B3] Aghakhani N. (2022). Relationship between mild traumatic brain injury and the gut microbiome: a scoping review. *J. Neurosci. Res.* 100 827–834. 10.1002/jnr.25004 34964504

[B4] Alharthi A. Alhazmi S. Alburae N. Bahieldin A. (2022). The human gut microbiome as a potential factor in autism spectrum disorder. *Int. J. Mol. Sci.* 23:1363. 10.3390/ijms23031363 35163286 PMC8835713

[B5] Alvarez E. O. (2009). The role of histamine on cognition. *Behav. Brain Res.* 199 183–189. 19126417 10.1016/j.bbr.2008.12.010

[B6] Baker D. Van den Beek M. Blankenberg D. (2016). The galaxy platform for accessible, reproducible and collaborative biomedical analyses: 2016 update. *Acids Res.* 44 W3–W10. 10.1093/nar/gkw343 27137889 PMC4987906

[B7] Batut B. Gravouil K. Defois C. Hiltemann S. Brugère J.-F. Peyretaillade E. (2018). ASaiM: a galaxy-based framework to analyze microbiota data. *Gigascience* 7:giy057. 10.1093/gigascience/giy057 29790941 PMC6007547

[B8] Bellomo G. Piscopo P. Corbo M. Pupillo E. Stipa G. Beghi E. (2022). A systematic review on the risk of neurodegenerative diseases and neurocognitive disorders in professional and varsity athletes. *Neurol. Sci.* 43 6667–6691. 10.1007/s10072-022-06319-x 35976476 PMC9663371

[B9] Berger A. A. Winnick A. Welschmeyer A. Kaneb A. Berardino K. Cornett E. M. (2020). Istradefylline to treat patients with Parkinson’s disease experiencing “off” episodes: a comprehensive review. *Neurol. Int.* 12 109–129. 10.3390/neurolint12030017 33302331 PMC7768423

[B10] Børsheim E. Bui Q.-U. T. Wolfe R. R. (2007). Plasma amino acid concentrations during late rehabilitation in patients with traumatic brain injury. *Arch. Phys. Med. Rehabil.* 88 234–238. 10.1016/j.apmr.2006.11.003 17270522

[B11] Brenner L. A. Stamper C. E. Hoisington A. J. Stearns-Yoder K. A. Stanislawksi M. A. Brostow D. P. (2020). Microbial diversity and community structures among those with moderate to severe TBI: a united states-veteran microbiome project study. *J. Head Trauma Rehabil.* 35 332–341. 10.1097/HTR.0000000000000615 32881767

[B12] Brenner L. A. Stearns-Yoder K. A. Hoffberg A. S. Penzenik M. E. Starosta A. J. Hernández T. D. (2017). Growing literature but limited evidence: a systematic review regarding prebiotic and probiotic interventions for those with traumatic brain injury and/or posttraumatic stress disorder. *Brain Behav. Immun.* 65 57–67. 28606462 10.1016/j.bbi.2017.06.003

[B13] Bruno J. F. Song J. Xu Y. Berelowitz M. (1993). Regulation of hypothalamic preprogrowth hormone-releasing factor messenger ribonucleic acid expression in food-deprived rats: a role for histaminergic neurotransmission. *Endocrinology* 133 1377–1381. 10.1210/endo.133.3.8103451 8103451

[B14] Cacabelos R. (1991). “Histaminergic regulation of the neuroendocrine system (NES),” in *Histaminergic Neurons: Morphology and Function*, eds Watanabe T. Wada H. (CRC Press: New York), 241–270.

[B15] Cederberg D. Siesjö P. (2010). What has inflammation to do with traumatic brain injury? *Childs. Nerv. Syst.* 26 221–226. 19940996 10.1007/s00381-009-1029-x

[B16] Chatzikonstantinou S. Gioula G. Kimiskidis V. K. McKenna J. Mavroudis I. Kazis D. (2021). The gut microbiome in drug-resistant epilepsy. *Epilepsia Open* 6 28–37. 33681645 10.1002/epi4.12461 PMC7918308

[B17] Chen L. Wang D. Garmaeva S. Kurilshikov A. Vich Vila A. Gacesa R. (2021). The long-term genetic stability and individual specificity of the human gut microbiome. *Cell* 184 2302–2315.e12. 33838112 10.1016/j.cell.2021.03.024

[B18] Corps K. N. Roth T. L. McGavern D. B. (2015). Inflammation and neuroprotection in traumatic brain injury. *JAMA Neurol.* 72 355–362. 25599342 10.1001/jamaneurol.2014.3558 PMC5001842

[B19] Corrigan J. D. Yang J. Singichetti B. Manchester K. Bogner J. (2018). Lifetime prevalence of traumatic brain injury with loss of consciousness. *Inj. Prev.* 24 396–404. 28848057 10.1136/injuryprev-2017-042371

[B20] Dai Z.-L. Wu G. Zhu W.-Y. (2011). Amino acid metabolism in intestinal bacteria: links between gut ecology and host health. *Front. Biosci.* 16 1768–1786. 10.2741/3820 21196263

[B21] Dinan K. Dinan T. G. (2022). Gut microbes and neuropathology: is there a causal nexus? *Pathogens* 11:796. 10.3390/pathogens11070796 35890040 PMC9319901

[B22] Durham W. J. Foreman J. P. Randolph K. M. Danesi C. P. Spratt H. Masel B. D. (2017). hypoaminoacidemia characterizes chronic traumatic brain injury. *J. Neurotrauma* 34 385–390. 27178787 10.1089/neu.2015.4350

[B23] Elfil M. Kamel S. Kandil M. Koo B. B. Schaefer S. M. (2020). Implications of the gut microbiome in Parkinson’s disease. *Mov. Disord.* 35 921–933. 32092186 10.1002/mds.28004

[B24] Esterov D. Sperl M. A. Hines E. A. Kinzelman Vesely E. A. Brown A. W. (2023). Association between traumatic brain injury and increased risk of stroke: a systematic review and meta-analysis. *J. Head Trauma Rehabil.* 38 E44–E55. 36594863 10.1097/HTR.0000000000000785 PMC9813869

[B25] Faith J. J. Guruge J. L. Charbonneau M. Subramanian S. Seedorf H. Goodman A. L. (2013). The long-term stability of the human gut microbiota. *Science* 341:1237439. 10.1126/science.1237439 PMC3791589 23828941

[B26] Feurle J. Espinosa E. Eckstein S. Pont F. Kunzmann V. Fournié J.-J. (2002). *Escherichia coli* produces phosphoantigens activating human γδ T cells. *J. Biol. Chem.* 277 148–154. 11675382 10.1074/jbc.M106443200

[B27] Frost F. Kacprowski T. Rühlemann M. Pietzner M. Bang C. Franke A. (2021). Long-term instability of the intestinal microbiome is associated with metabolic liver disease, low microbiota diversity, diabetes mellitus and impaired exocrine pancreatic function. *Gut* 70 522–530. 10.1136/gutjnl-2020-322753 33168600 PMC7873430

[B28] George A. K. Behera J. Homme R. P. Tyagi N. Tyagi S. C. Singh M. (2021). Rebuilding microbiome for mitigating traumatic brain injury: importance of restructuring the gut-microbiome-brain axis. *Mol. Neurobiol.* 58 3614–3627. 10.1007/s12035-021-02357-2 33774742 PMC8003896

[B29] Gomes C. V. Kaster M. P. Tomé A. R. Agostinho P. M. Cunha R. A. (2011). Adenosine receptors and brain diseases: neuroprotection and neurodegeneration. *Biochim. Biophys. Acta* 1808 1380–1399. 21145878 10.1016/j.bbamem.2010.12.001

[B30] Haas H. Panula P. (2003). The role of histamine and the tuberomamillary nucleus in the nervous system. *Nat. Rev. Neurosci.* 4 121–130. 12563283 10.1038/nrn1034

[B31] Haas H. L. Sergeeva O. A. Selbach O. (2008). Histamine in the nervous system. *Physiol. Rev.* 88 1183–1241. 18626069 10.1152/physrev.00043.2007

[B32] Hanscom M. Loane D. J. Shea-Donohue T. (2021). Brain-gut axis dysfunction in the pathogenesis of traumatic brain injury. *J. Clin. Invest.* 131:e143777. 10.1172/JCI143777 34128471 PMC8203445

[B33] Holmes M. Flaminio Z. Vardhan M. Xu F. Li X. Devinsky O. (2020). Cross talk between drug-resistant epilepsy and the gut microbiome. *Epilepsia* 61 2619–2628. 10.1111/epi.16744 33140419

[B34] Johnson D. Letchumanan V. Thurairajasingam S. Lee L.-H. (2020). A revolutionizing approach to autism spectrum disorder using the microbiome. *Nutrients* 12:1983. 10.3390/nu12071983 32635373 PMC7400420

[B35] Kigerl K. A. Hall J. C. E. Wang L. Mo X. Yu Z. Popovich P. G. (2016). Gut dysbiosis impairs recovery after spinal cord injury. *J. Exp. Med.* 213 2603–2620. 27810921 10.1084/jem.20151345 PMC5110012

[B36] Kim J. M. Rim J. H. Kim D. H. Kim H. Y. Choi S. K. Kim D. Y. (2021). P157 microbiome analysis reveals that ralstonia is responsible for decreased renal function in patients with ulcerative colitis. *J. Crohns. Colitis* 15 S236–S238. 10.1002/ctm2.322 33784015 PMC7933010

[B37] Kopylova E. Noé L. Touzet H. (2012). SortMeRNA: fast and accurate filtering of ribosomal RNAs in metatranscriptomic data. *Bioinformatics* 28 3211–3217. 10.1093/bioinformatics/bts611 23071270

[B38] Lambert M. Sheldrake E. Deneault A.-A. Wheeler A. Burke M. Scratch S. (2022). Depressive symptoms in individuals with persistent postconcussion symptoms: a systematic review and meta-analysis. *JAMA Netw. Open* 5:e2248453. 10.1001/jamanetworkopen.2022.48453 PMC9857135 36574246

[B39] Lin W. Xu L. Zheng Y. An S. Zhao M. Hu W. (2023). Whole-brain mapping of histaminergic projections in mouse brain. *Proc. Natl. Acad. Sci. U. S. A.* 120:e2216231120. 10.1073/pnas.2216231120 36976764 PMC10083611

[B40] Lum G. R. Olson C. A. Hsiao E. Y. (2020). Emerging roles for the intestinal microbiome in epilepsy. *Neurobiol. Dis.* 135:104576. 10.1016/j.nbd.2019.104576 31445165

[B41] Lusardi T. A. (2009). Adenosine neuromodulation and traumatic brain injury. *Curr. Neuropharmacol.* 7 228–237. 20190964 10.2174/157015909789152137 PMC2769006

[B42] Maeda K. Frohman L. A. (1978). Dissociation of systemic and central effects of neurotensin on the secretion of growth hormone, prolactin, and thyrotropin. *Endocrinology* 103 1903–1909. 10.1210/endo-103-5-1903 107023

[B43] Martin M. (2011). Cutadapt removes adapter sequences from high-throughput sequencing reads. *EMBnet.J* 17 10–12. 10.1089/cmb.2017.0096 28715235

[B44] Mehta S. Crane M. Leith E. Batut B. Hiltemann S. Arntzen M. Ø (2021). ASaiM-MT: a validated and optimized ASaiM workflow for metatranscriptomics analysis within Galaxy framework. *F1000Res* 10:103. 10.12688/f1000research.28608.2 34484688 PMC8383124

[B45] Metsalu T. Vilo J. (2015). ClustVis: a web tool for visualizing clustering of multivariate data using Principal Component Analysis and heatmap. *Nucleic Acids Res.* 43 W566–W570. 10.1093/nar/gkv468 25969447 PMC4489295

[B46] Mills R. H. Dulai P. S. Vázquez-Baeza Y. Sauceda C. Daniel N. Gerner R. R. (2022). Multi-omics analyses of the ulcerative colitis gut microbiome link *Bacteroides* vulgatus proteases with disease severity. *Nat. Microbiol.* 7 262–276. 10.1038/s41564-021-01050-3 35087228 PMC8852248

[B47] Moro J. Tomé D. Schmidely P. Demersay T.-C. Azzout-Marniche D. (2020). Histidine: a systematic review on metabolism and physiological effects in human and different animal species. *Nutrients* 12:1414. 10.3390/nu12051414 32423010 PMC7284872

[B48] Mossberg K. A. Durham W. J. Zgaljardic D. J. Gilkison C. R. Danesi C. P. Sheffield-Moore M. (2017). Functional changes after recombinant human growth hormone replacement in patients with chronic traumatic brain injury and abnormal growth hormone secretion. *J. Neurotrauma* 34 845–852. 10.1089/neu.2016.4552 27627580 PMC5314981

[B49] Nelson L. D. Temkin N. R. Barber J. Brett B. L. Okonkwo D. O. McCrea M. A. (2023). Functional recovery, symptoms, and quality of life 1 to 5 years after traumatic brain injury. *JAMA Netw Open* 6:e233660. 10.1001/jamanetworkopen.2023.3660 PMC10028488 36939699

[B50] Nicholson S. E. Watts L. T. Burmeister D. M. Merrill D. Scroggins S. Zou Y. (2019). Moderate traumatic brain injury alters the gastrointestinal microbiome in a time-dependent manner. *Shock* 52 240–248. 10.1097/SHK.0000000000001211 29953417

[B51] Opeyemi O. M. Rogers M. B. Firek B. A. Janesko-Feldman K. Vagni V. Mullett S. J. (2021). Sustained dysbiosis and decreased fecal short-chain fatty acids after traumatic brain injury and impact on neurologic outcome. *J. Neurotrauma* 38 2610–2621. 10.1089/neu.2020.7506 33957773 PMC8403202

[B52] Passani M. B. Bacciottini L. Mannaioni P. F. Blandina P. (2000). Central histaminergic system and cognition. *Neurosci. Biobehav. Rev.* 24 107–113. 10654665 10.1016/s0149-7634(99)00053-6

[B53] Pathare N. Sushilkumar S. Haley L. Jain S. Osier N. N. (2020). The impact of traumatic brain injury on microbiome composition: a systematic review. *Biol. Res. Nurs.* 22 495–505. 10.1177/1099800420943961 32720519

[B54] Peterman M. G. (1924). The ketogenic diet in the treatment of epilepsy: a preliminary report. *Am. J. Dis. Child.* 28 28–33.

[B55] Peterson A. B. Thomas K. E. (2021). Incidence of Nonfatal Traumatic Brain Injury-Related Hospitalizations - United States, 2018. *MMWR Morb. Mortal. Wkly. Rep.* 70 1664–1668. 34855719 10.15585/mmwr.mm7048a3 PMC8641562

[B56] Read T. B. I. (n.d.). *TBI Data. cdc.gov.* Available online at: https://www.cdc.gov/traumaticbraininjury/data/tbi-deaths.html (accessed November 19, 2023).

[B57] Rice M. W. Pandya J. D. Shear D. A. (2019). Gut microbiota as a therapeutic target to ameliorate the biochemical, neuroanatomical, and behavioral effects of traumatic brain injuries. *Front. Neurol.* 10:875. 10.3389/fneur.2019.00875 31474930 PMC6706789

[B58] Rogers G. B. Keating D. J. Young R. L. Wong M.-L. Licinio J. Wesselingh S. (2016). From gut dysbiosis to altered brain function and mental illness: mechanisms and pathways. *Mol. Psychiatry* 21 738–748. 27090305 10.1038/mp.2016.50 PMC4879184

[B59] Sarkar A. Harty S. Lehto S. M. Moeller A. H. Dinan T. G. Dunbar R. I. M. (2018). The microbiome in psychology and cognitive neuroscience. *Trends Cogn. Sci.* 22 611–636. 29907531 10.1016/j.tics.2018.04.006

[B60] Sochocka M. Donskow-Łysoniewska K. Diniz B. S. Kurpas D. Brzozowska E. Leszek J. (2019). The gut microbiome alterations and inflammation-driven pathogenesis of Alzheimer’s disease-a critical review. *Mol. Neurobiol.* 56 1841–1851. 10.1007/s12035-018-1188-4 29936690 PMC6394610

[B61] Soriano S. Curry K. Sadrameli S. S. Wang Q. Nute M. Reeves E. (2022). Alterations to the gut microbiome after sport-related concussion in a collegiate football players cohort: a pilot study. *Brain Behav Immun Health* 21:100438. 10.1016/j.bbih.2022.100438 35284846 PMC8914332

[B62] Sun M. Ma K. Wen J. Wang G. Zhang C. Li Q. (2020). A review of the brain-gut-microbiome axis and the potential role of microbiota in Alzheimer’s disease. *J. Alzheimers. Dis.* 73 849–865. 31884474 10.3233/JAD-190872

[B63] Sundman M. H. Chen N.-K. Subbian V. Chou Y.-H. (2017). The bidirectional gut-brain-microbiota axis as a potential nexus between traumatic brain injury, inflammation, and disease. *Brain Behav. Immun.* 66 31–44. 10.1016/j.bbi.2017.05.009 28526435

[B64] Tai K. Leland E. M. Seal S. M. Schneider A. L. C. Rowan N. R. Kamath V. (2022). Olfactory dysfunction following moderate to severe traumatic brain injury: a systematic review and meta-analysis. *Neuropsychol. Rev.* 33 717–732. 10.1007/s11065-022-09563-2 36070126 PMC10040093

[B65] Tashiro M. Mochizuki H. Iwabuchi K. Sakurada Y. Itoh M. Watanabe T. (2002). Roles of histamine in regulation of arousal and cognition: functional neuroimaging of histamine H1 receptors in human brain. *Life Sci.* 72 409–414. 10.1016/s0024-3205(02)02276-2 12467881

[B66] Treangen T. J. Wagner J. Burns M. P. Villapol S. (2018). Traumatic brain injury in mice induces acute bacterial dysbiosis within the fecal microbiome. *Front. Immunol.* 9:2757. 10.3389/fimmu.2018.02757 30546361 PMC6278748

[B67] Truong D. T. Franzosa E. A. Tickle T. L. Scholz M. Weingart G. Pasolli E. (2015). MetaPhlAn2 for enhanced metagenomic taxonomic profiling. *Nat. Methods* 12 902–903. 10.1038/nmeth.3589 26418763

[B68] Udayappan S. D. Kovatcheva-Datchary P. Bakker G. J. Havik S. R. Herrema H. Cani P. D. (2017). Intestinal Ralstonia pickettii augments glucose intolerance in obesity. *PLoS One* 12:e0181693. 10.1371/journal.pone.0181693 29166392 PMC5699813

[B69] Urban R. J. Pyles R. B. Stewart C. J. Ajami N. Randolph K. M. Durham W. J. (2020). Altered fecal microbiome years after traumatic brain injury. *J. Neurotrauma* 37 1037–1051. 10.1089/neu.2019.6688 31868094

[B70] Wang C. Zhao J. Zhang H. Lee Y.-K. Zhai Q. Chen W. (2021). Roles of intestinal *bacteroides* in human health and diseases. *Crit. Rev. Food Sci. Nutr.* 61 3518–3536. 10.1080/10408398.2020.1802695 32757948

[B71] Wolfe A. J. (2015). Glycolysis for microbiome generation. *Microbiol. Spectr.* 3 10.1128/microbiolspec.MBP-0014-2014 26185089 PMC4507297

[B72] Wright C. Shin J. H. Rajpurohit A. Deep-Soboslay A. Collado-Torres L. Brandon N. J. (2017). Altered expression of histamine signaling genes in autism spectrum disorder. *Transl. Psychiatry* 7:e1126. 10.1038/tp.2017.87 28485729 PMC5534955

[B73] Wright T. Urban R. Durham W. Dillon E. L. Randolph K. M. Danesi C. (2020). Growth hormone alters brain morphometry, connectivity, and behavior in subjects with fatigue after mild traumatic brain injury. *J. Neurotrauma* 37 1052–1066. 10.1089/neu.2019.6690 31797721 PMC7185353

[B74] Wright T. J. Elliott T. R. Randolph K. M. Pyles R. B. Masel B. E. Urban R. J. (in review). Prevalence of fatigue and cognition after traumatic brain injury. *PLoS One*10.1371/journal.pone.0300910 PMC10959386 38517903

[B75] Yanckello L. M. Chang Y.-H. Sun M. Chlipala G. Green S. J. Lei Z. (2022a). Inulin supplementation prior to mild traumatic brain injury mitigates gut dysbiosis, and brain vascular and white matter deficits in mice. *Front. Microbiomes* 1:986951. 10.3389/frmbi.2022.986951 36756543 PMC9903356

[B76] Yanckello L. M. Fanelli B. McCulloch S. Xing X. Sun M. Hammond T. C. (2022b). Inulin supplementation mitigates gut dysbiosis and brain impairment induced by mild traumatic brain injury during chronic phase. *J. Cell Immunol.* 4 50–64. 10.33696/immunology.4.132 35611116 PMC9126115

[B77] Yi L.-J. Tian X. Shi B. Pi Y.-P. Chen W.-Q. (2019). Early enteral nutrition supplemented with probiotics improved the clinical outcomes in severe head injury: some promising findings from Chinese patients. *Medicine* 98: e15426. 10.1097/MD.0000000000015426 PMC6831228 31027144

[B78] Yoshikawa T. Nakamura T. Yanai K. (2021). Histaminergic neurons in the tuberomammillary nucleus as a control centre for wakefulness. *Br. J. Pharmacol.* 178 750–769. 32744724 10.1111/bph.15220

[B79] Yuen K. C. J. Masel B. E. Reifschneider K. L. Sheffield-Moore M. Urban R. J. Pyles R. B. (2020). Alterations of the GH/IGF-I axis and gut microbiome after traumatic brain injury: a new clinical syndrome? *J. Clin. Endocrinol. Metab.* 105:dgaa398. 10.1210/clinem/dgaa398 32585029

[B80] ZhaoY.ZhouY.-G.ChenJ.-F. (2023). Targeting the adenosine A2A receptor for neuroprotection and cognitive improvement in traumatic brain injury and Parkinson’s disease. *Chin. J. Traumatol.* Online ahead of print. 10.1016/j.cjtee.2023.08.003 37679245

