# Supplementary material for: The altered TBI fecal microbiome is stable and functionally distinct
Source: Front Mol Neurosci. 2024 Mar 13;17:1341808. doi: 10.3389/fnmol.2024.1341808 (PMC10965628; doi:10.3389/fnmol.2024.1341808)
Supplement: Supplementary file 1 [file Table_1.docx]

Supplemental Table 1. Novel qPCR targets for metagenomic analyses.

| **Primer** | **Forward** | **Reverse** | **Target** |
| --- | --- | --- | --- |
| *Bacteroides cellulosilyticus* | ATGATTATAGAGGCTGATGAAT | TAGGTTCCAGTTCGTTGA | DNA gyrase A |
| *Corynebacterium propinquum* | CGTGCCGAAGAACTCAAC | CTCGTCCAGCCCTAACAC | DNA polymerase III subunit epsilon |
| *Corynebacterium spp* | GGGTAATGGCCTACCAAG | CCGTATCTCAGTCCCAATG | 16s |
| *Propionibacterium acnes* | TTGCCAACAAATTGACTTTA | GGCTGTTCTTGGTAGAAG | RNA methyltransferase |
| *Ralstonia pickettii* | CGGCAAGGAAGAAGACTT | AGCACGGTCTTGTTCTTG | DNA gyrase B |
| *Ralstonia pickettii* | GGCACCATCAAGAAGACCAAT | GCCGAACATCACACCGAT | recombinase A |

Based on ongoing findings from human FMB analyses, additional targets beyond the original 96 were developed and employed for these assays using the primers and gene targets as noted above.
